# BAFF Receptor mAb Treatment Ameliorates Development and Progression of Atherosclerosis in Hyperlipidemic ApoE^−/−^ Mice

**DOI:** 10.1371/journal.pone.0060430

**Published:** 2013-04-03

**Authors:** Tin Kyaw, Peng Cui, Christopher Tay, Peter Kanellakis, Hamid Hosseini, Edgar Liu, Antonius G. Rolink, Peter Tipping, Alex Bobik, Ban-Hock Toh

**Affiliations:** 1 Vascular Biology and Atherosclerosis Laboratory Baker IDI Heart and Diabetes Institute, Victoria, Australia; 2 Centre for Inflammatory Diseases Department of Medicine, Southern Clinical School, Faculty of Medicine, Nursing and Health Sciences, Monash University, Victoria, Australia; 3 Developmental and Molecular Immunology, Department of Biomedicine, University of Basel, Basel, Switzerland; Charité, Campus Benjamin Franklin, Germany

## Abstract

**Aims:**

Option to attenuate atherosclerosis by depleting B2 cells is currently limited to anti-CD20 antibodies which deplete all B-cell subtypes. In the present study we evaluated the capacity of a monoclonal antibody to B cell activating factor-receptor (BAFFR) to selectively deplete atherogenic B2 cells to prevent both development and progression of atherosclerosis in the ApoE^−/−^ mouse.

**Methods and Results:**

To determine whether the BAFFR antibody prevents atherosclerosis development, we treated ApoE^−/−^ mice with the antibody while feeding them a high fat diet (HFD) for 8 weeks. Mature CD93^−^ CD19^+^ B2 cells were reduced by treatment, spleen B-cell zones disrupted and spleen CD20 mRNA expression decreased while B1a cells and non-B cells were spared. Atherosclerosis was ameliorated in the hyperlipidemic mice and CD19^+^ B cells, CD4^+^ and CD8^+^ T cells were reduced in atherosclerotic lesions. Expressions of proinflammatory cytokines, IL1β, TNFα, and IFNγ in the lesions were also reduced, while MCP1, MIF and VCAM-1 expressions were unaffected. Plasma immunoglobulins were reduced, but MDA-oxLDL specific antibodies were unaffected. To determine whether anti-BAFFR antibody ameliorates progression of atherosclerosis, we first fed ApoE^−/−^ mice a HFD for 6 weeks, and then instigated anti-BAFFR antibody treatment for a further 6 week-HFD. CD93^−^ CD19^+^ B2 cells were selectively decreased and atherosclerotic lesions were reduced by this treatment.

**Conclusion:**

Anti-BAFFR monoclonal antibody selectively depletes mature B2 cells while sparing B1a cells, disrupts spleen B-cell zones and ameliorates atherosclerosis development and progression in hyperlipidemic ApoE^−/−^ mice. Our findings have potential for clinical translation to manage atherosclerosis-based cardiovascular diseases.

## Introduction

Atherosclerosis-based heart attacks and strokes are the leading causes of global deaths [1]. The lethal complications of atherosclerosis arise from thrombotic occlusion of ruptured atherosclerotic plaques that develop as a consequence of inflammation initiated by lipid entry into the arterial wall. Lipid-reduction by the statins in atherosclerosis management is effective in only one-third of patients [2]. There is therefore an urgent need to develop additional therapeutic strategies to reduce the inflammatory component of atherosclerosis in the management of atherosclerosis-based cardiovascular disease.

We have previously reported that B cell depletion by an anti-CD20 monoclonal antibody potently reduces atherosclerotic lesions. The treatment not only ameliorates atherosclerosis development but is also effective in reducing established atherosclerotic lesions in hyperlipidemic ApoE^−/−^ mice [3]. The capacity of B cell depletion by an anti-CD20 monoclonal antibody to ameliorate atherosclerosis was also independently reported by Ait-Oufella et al in LDLR^−/−^ mice [4]. These findings are consistent with the amelioration of mouse and human autoimmune diseases by B cell depletion therapy with anti-CD20 monoclonal antibody [5], [6]. The strategy of B cell depletion with anti-CD20 monoclonal antibody is currently successfully used in the treatment of rheumatoid arthritis [7] and being increasing explored for the treatment of other human autoimmune diseases [8], [9].

We identified B2 lymphocytes as the atherogenic population by their adoptive transfer to B cell deficient (µMT) mice as well as to lymphocyte-deficient mice [3]. Given that B2 lymphocytes are dependent on the interaction of BAFF (B cell activation factor of the TNF family) with BAFF-receptor (BAFFR) for their survival and maturation [10], [11], we crossed BAFFR-deficient mice to ApoE^−/−^ mice and examined how BAFFR deficiency affected development of atherosclerosis. We found that these double knockout mice also displayed ameliorated atherosclerosis [12]. Our findings were also supported by the report that LDL receptor deficient mice rendered chimeric by transplantation of bone marrow from BAFFR deficient mice also displayed reduced atherosclerosis [13]. The established atherogenicity of B2 cells stands in stark contrast to that of innate-like B1a cells that we have reported to be atheroprotective by the secretion of natural IgM that scavenges apoptotic cells [14]. We have reviewed the contrasting properties of atherogenic B2 cells to those of atheroprotective B1a cells [14], [15].

BAFF is widely expressed by immune cells, primarily macrophages and dendritic cells and binds to 3 receptors, BCMA (B-cell maturation antigen/TNFRSF17), TACI (transmembrane activator and calcium-modulator and cyclophilin ligand interactor; TNFRSF13B) and BAFFR (BAFF-receptor; TNFRSF13C) [16]. Whilst BCMA and TACI is differentially expressed on different B cell subsets, BAFFR is expressed by all immature and mature B cells with highest expression in mature B cells [17]. BAFFR expression in mice and in humans correlates with positive selection of immature B cells [18]. BAFFR is an appealing therapeutic target to selectively deplete mature B2 cells in B- cell targeted therapy because in contrast to BCMA and TACI that bind both BAFF and its homolog APRIL, BAFFR binds exclusively to BAFF. The report that a single injection of BAFFR monoclonal antibody that prevents BAFF binding drastically reduced mature B cells without affecting B1a cells [19] prompted us to explore the therapeutic potential of this monoclonal antibody to BAFFR to attenuate atherosclerosis in the hyperlipidemic ApoE^−/−^ mouse. Using this approach we found that anti-BAFFR monoclonal antibody prevented atherosclerosis development and also ameliorated the progression of established atherosclerosis. This novel therapeutic strategy specifically targeting B2 cells in atherosclerosis may have potential for clinical translation.

## Materials and Methods

### Ethic Statement

All experiments approved by the Alfred Medical Research and Education Precinct (AMREP) Animal Ethics Committee (approved project number E/1278/2012/B) were carried out at the Precinct Animal Centre, AMREP, Prahran, Victoria, Australia.

### Anti-BAFFR Monoclonal Antibody

The rat anti-mouse BAFFR monoclonal IgG antibody used to deplete mature B2 cells has been described previously [19]. An intravenous injection of 0.5 mg antibody was given to deplete B2 cells and subsequent similar doses were scheduled fortnightly to maintain the depletion of mature B2 cells in ApoE^−/−^ C57Bl/6 mice. Rat IgG (Sigma) was used as control antibody.

### Animals and Experiment Design

Male 6–8 weeks-old C57Bl/6 mice deficient in ApoE gene were fed a high fat diet (HFD) comprised of 21% fat and 0.15% cholesterol (Specialty Feeds, Glen Forrest, Western Australia) and sterile water that were given ad libitum throughout the experiment. In the prevention study, antibody treatment was given to ApoE^−/−^ mice at the start of 8 week-HFD, and in the intervention study, mice were fed a HFD for 6 weeks to generate atherosclerosis, followed by antibody treatment for a further 6 weeks to investigate therapeutic potential of the antibody in established atherosclerosis. At the end of experiment, mice were euthanized in slow-filled carbon dioxide chamber for three minutes and after assessing lack of respiration and faded eye colour to confirm unconsciousness blood was extracted by cardiac puncture in EDTA-containing syringes.

### Tissue Collection

Plasma were separated and kept in a -80C freezer for lipid and immunoglobulin analyses. Blood, peritoneal fluid, peripheral lymph nodes (inguinal and auxillary) and spleens were processed into single cell suspensions for FACS analysis. The aortic arch and thoracic aorta were snap-frozen in liquid nitrogen and kept in a −80°C freezer for mRNA expression analysis. Aortic roots and spleen were embedded in OCT medium (Tissue-Tek) and kept at −80°C for subsequent atherosclerosis assessment and immunohistochemical analysis.

### Lymphocyte Assessment

The following fluorochrome-conjugated anti-mouse antibodies were used: anti-CD22 (PE; BD Biosciences), anti-CD5 (APC; BD Biosciences), anti-CD11b (APC-Cy7; BD Biosciences), anti-CD93 (PE-Cy7; BD Biosciences), anti-CD4 (Pacific Blue; Invitrogen), anti-CD8 (PerCP; BD Biosciences), anti-NK1.1 (PE-Cy7, BD Biosciences), anti-TCRβ (FITC; BD Biosciences), anti-CD25 (APC-Cy7; BD Biosciences), anti-foxp3 (PE; eBioscience). Cells isolated from blood, peritoneal fluid, peripheral lymph nodes and spleens were stained for lymphocyte assessment as described before [12]. FACS data were acquired in CantoII flow cytometer (BD Biosciences) and analyzed using FACS Diva software (BD Biosciences).

### Atherosclerosis Assessment

Atherosclerotic lesions at aortic sinuses were assessed by oil red-O stained lipid accumulation as described [20]. In addition to lipid accumulation, atherosclerosis was also assessed by measuring intimal atherosclerotic lesion areas using image analysis software (Optimas, South Australia, Australia) and light microscopy.

### Immunohistochemical Analysis at Atherosclerotic Lesions

Macrophages, the proinflammatory cytokine IL1β, CD19^+^ B cells, CD4^+^ and CD8^+^ T cells were assessed by immunohistochemical methods. Briefly, rabbit anti-mouse IL1β (Abcam, UK), rat anti-mouse CD68 (Serotec, Raleigh, NC), rat anti-mouse CD19 (BD Biosciences ), rat anti-mouse CD4 (BD Biosciences) and rat anti-mouse CD8 (BD Biosciences) antibodies were used to detect IL1β, CD68^+^ macrophages, CD19^+^ B cells, CD4^+^ and CD8^+^ T cells in atherosclerotic lesions [12].

After fixing in acetone, quenching endogenous peroxidise and non-specific blocking with 10% normal horse serum, frozen sections of the aortic sinus were incubated with primary antibodies. Biotinylated goat anti-rat or anti-rabbit immunoglobulins were used in secondary antibody incubation. VECTORSTAIN elite Avidin-Biotin Complex kit (Vector laboratories) and 3,3′-Diaminobenzidine (DAB) were used to generate brown coloration. After counterstaining with haematoxylin and dehydration, slides were mounted with DEPEX.

Quantification of IL1β and macrophage accumulation was assessed by DAB stained area using Optimas software and expressed as % total lesion area [12]. Cells stained with CD19, CD4 and CD8 were manually counted under light microscopy and corrected to total lesion areas as quantified by Optima software [12].

### Plasma Lipid Analysis

Plasma lipid profiles (LDL-Cholesterol, HDL-Cholesterol, total cholesterol and triglycerides) were assessed as described before [20].

### ELISA Measurement

Plasma levels of total and MDA-LDL specific antibodies were determined by enzyme-linked immunosorbent assay as described before [21] and plasma BAFF levels were measured according to manufacturer’s instructions using Mouse BAFF/BLyS/TNFSF13B Immunoassay (R&D systems).

### Spleen Architecture Analysis

Micro-architecture of frozen spleen sections was visualised using confocal microscopy. After fixing in acetone and blocking autofluorescence with 50 mM NH_4_Cl, B cells in frozen spleen sections were stained by FITC-labelled rat anti-mouse B220 (BD Biosciences). For T cells, sections were first incubated with purified hamster anti-mouse CD3 (BD Biosciences), followed by goat anti-hamster secondary antibody conjugated with Alexa-Flor 546 (Molecular Probes). Nuclei were counterstained with 4′ 6-diamidino-2-phenylindole (DAPI). Images were scanned and generated by using Carl Zeiss Laser Scanning System LSM 510 and Zeiss LSM imaging software.

### Arterial mRNA Expression Analysis

RNeasy fibrous tissue mini kit (Qiagen) was used to extract total RNA from aortic arches according to manufacturer’s instruction. RNA quantity and integrity were determined using the MultiNA electrophoresis system (Shimadzu, Japan). mRNA expression was determined using single-step QuantiFast SYBR Green RT-PCR kit (Qiagen) on 7500 Fast Real-Time PCR system (Applied Biosystem). The target gene expression levels were analyzed using comparative cycle threshold method with 18S rRNA primers (Applied Biosystems). The primers used were as follows:

IL1β sense (S) 5′-CCACCTCAATGGACAGAATCTCAA-3′,

IL1β antisense (AS) 5′-GTCGTTGCTTGGTTCTCCTTGT-3′

TNFα (S) 5′-TCTCAGCCTCTTCTCATTCCT-3′,

TNFα (AS) 5′-ACTTGGTGGTTTGCTACGAC-3′;

IFNγ (S) 5′-AAGTTTGAGGTCAACAACCCAC-3′,

IFNγ (AS) 5′-GCTGGCAGAATTATTCTTATTGGG-3′;

TGFβ (S) 5′-AGCCCTGGATACCAACTATTGC-3′,

TGFβ (AS) 5′-TCCAACCCAGGTCCTTCCTAA-3′

MCP1 (S) 5′-CTCAGCCAGATGCAGTTAACG-3′,

MCP1 (AS) 5′-GGGTCAACTTCACATTCAAAGG-3′;

MIF (S) 5′-GGCAAGCCCGCACAGTAC-3′,

MIF (AS) 5′-ATCGTTCGTGCCGCTAAAAGT-3′.

VCAM-1 (S) 5′-AGAACCCAGACAGACAGTCC-3′

VCAM-1 (AS) 5′-GGATCTTCAGGGAATGAGTAGAC-3′.

CD20 (S) 5′-CTTATTCAAACTTCCAAGCCGT-3′,

CD20 (AS) 5′-GACAGAATGCCCAAGAACAC-3′

### Statistical Analysis

Statistical significance was calculated by 2-tailed Student t test or Mann-Whitney U test, depending on whether the data were normally distributed, as assessed by the Kolmogorov-Smirnov test using GraphPad Prism program. Results were presented as mean ±SEM. P values less than 0.05 were considered statistically significant.

## Results

### BAFFR Antibody Selectively Depletes Mature B cells in Hyperlipidemic ApoE^−/−^ Mice

**Figure 1:**
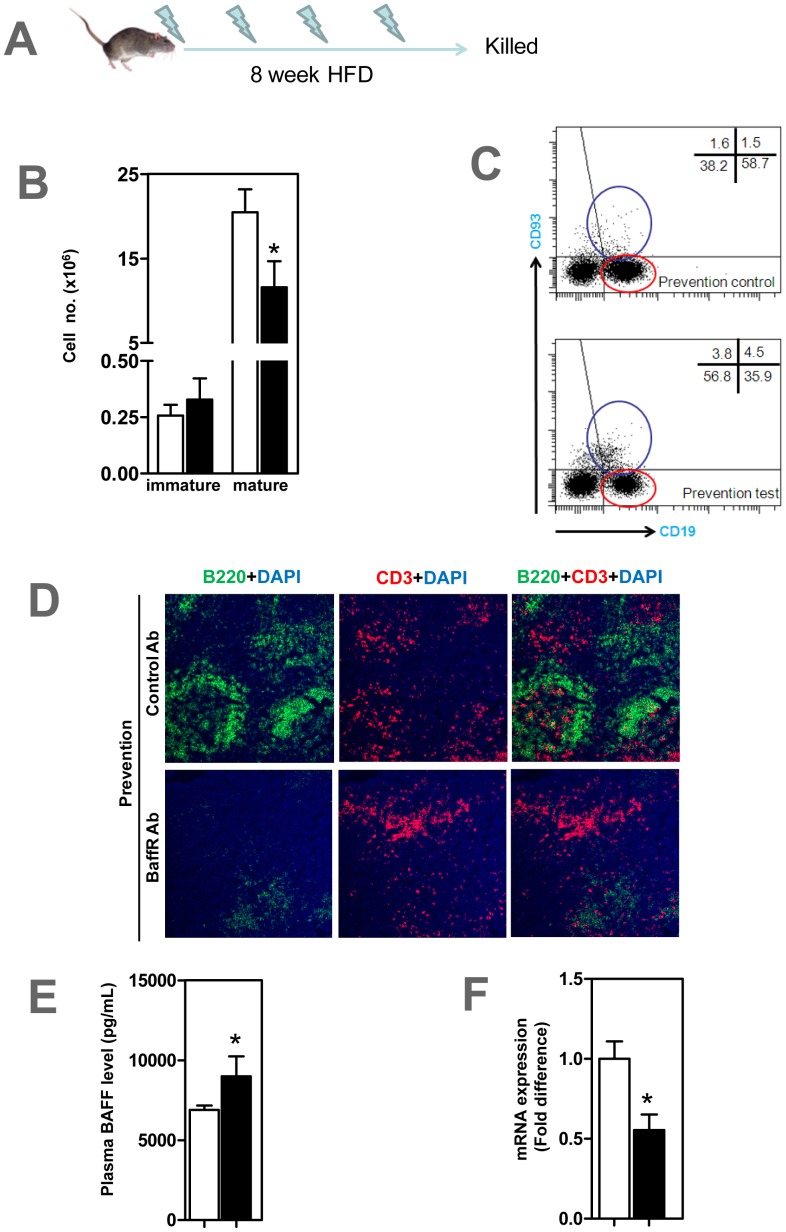
Mature B cell depletion in ApoE^−/−^ mice treated with anti-BAFFR antibody. (A) Male 6–8 weeks-old ApoE^−/−^ mice were given 0.5 mg anti-BAFFR antibody or control rat Ig fortnightly via tail vein injection while fed a high fat diet for 8 weeks. (B) CD93^−^ CD19^+^ mature B2 cells identified by FACS analysis significantly decreased in spleen, with CD93^+^ CD19^+^ immature B2 cells unaffected in anti-BAFFR-antibody treated mice. (C) Representative FACS showing mature and immature B cells in spleen stained with CD93 and CD19 antibodies. Blue circle represents CD93^+^ CD19^+^ immature B cells and red circle CD93^−^ CD19^+^ mature B cells (D) Confocal microscopy showing disrupted splenic B cell zones in treated, but not in control mice. B and T cells stained respectively with B220 and CD3 and counterstained with dapi (E) Plasma BAFF levels elevated in treated mice. (F) CD20 mRNA expression decreased in treated mice. n = 7–9 mice per group * p<0.05 □ control IgG treated mice; ▪ BAFFR-antibody treated mice.

At the end of prevention study (Figure 1A), mature CD93^−^ CD22^+^ B2 cells were depleted in blood (data not shown) and spleen (Figures 1B–C) of ApoE^−/−^ mice that received anti-BFFFR antibody compared to control group (P<0.05). Immature CD93^+^ CD22^+^ B cells in test mice tended to increase but this was not statistically significant (Figure 1B). Confocal microscopy showed that B-cell zones, not T-cell zones, in spleen were markedly disrupted in anti-BAFFR antibody treated ApoE^−/−^ mice with only low numbers of B220+ B cells (Figure 1D). The findings of increased plasma BAFF levels, by 30% ([P<0.05]; Figure 1E) and reduced CD20 expression in spleen by 45% ([P<0.05]; Figure 1F) is consistent with the B cell depletion following BAFFR antibody treatment. Collectively mature B2 cells that require BAFF-BAFFR interaction for their maintenance were reduced by 40% in ApoE^−/−^ mice that received anti-BAFFR antibody (Figure 1B).

### BAFFR Antibody Treatment does not Alter B1a B Cells, Non-B Cells and Plasma Lipid Profiles

**Figure 2:**
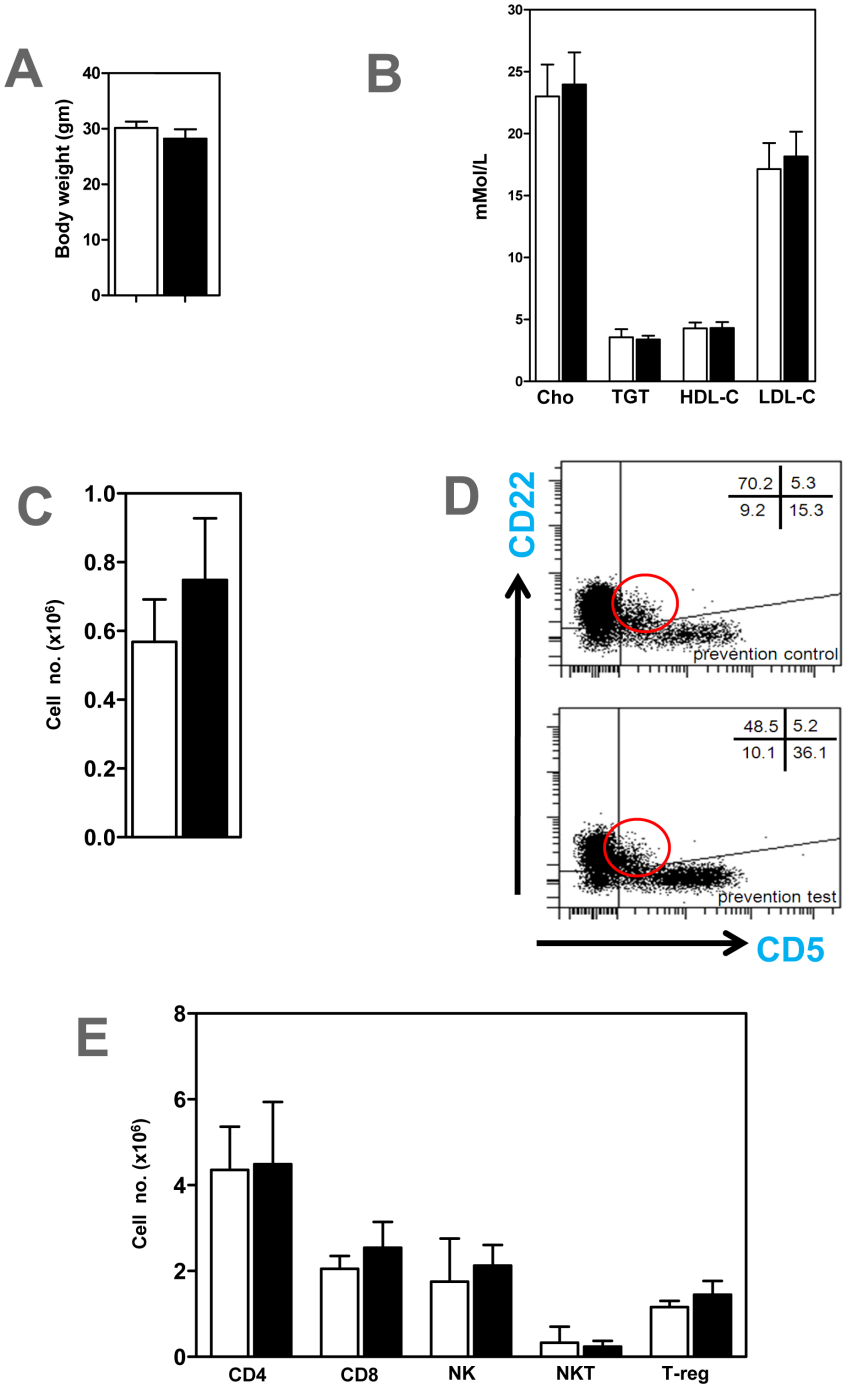
Body weight, plasma lipids, B1a and other lymphocytes unchanged in ApoE^−/−^ mice treated with anti-BAFFR antibody. A) Body weights, (B) Plasma lipids and (C) Peritoneal CD22^+^CD5^+^ B1a cells. (D) Peritoneal CD22^+^, CD5^+^ B1a B cells identified by FACS analysis. Red circle represents CD22^+^ CD5^+^ B1a cells (E) CD4^+^, CD8^+^, NK1.1^+^ (NK), NK1.1^+^ TCRβ^+^ (NKT) and CD4^+^ CD25^+^ foxp3^+^ regulatory T cells (T-reg) in spleens. n = 7–9 mice per each group □ control IgG treated mice; ▪ BAFFR-antibody treated mice.

Body weights (Figure 2A) and lipid profiles-LDL-Cholesterol, HDL-Cholesterol, total cholesterol and triglycerides (Figure 2B) determined in all experimental groups were unaffected by anti-BAFFR antibody treatment. Also anti-BAFFR antibody treatment did not affect the peritoneal CD22^+^ CD5^+^ B1a population (Figures 2C–D). FACS analysis on spleens indicated that CD4^+^ T cells, CD8^+^ T cells, NK and NKT cells were unaffected in anti-BAFFR antibody treated ApoE^−/−^ mice (Figure 2E). Similar findings were found in peripheral blood, peritoneal fluid and lymph nodes (data not shown). There was a trend for CD4^+^ CD25^+^ foxp3^+^ regulatory T cells to increase (Figure 2E), but this did not reach statistical significance (P>0.05).

### BAFFR Antibody-mediated Depletion of Mature B Cell Reduces Atherosclerosis

**Figure 3:**
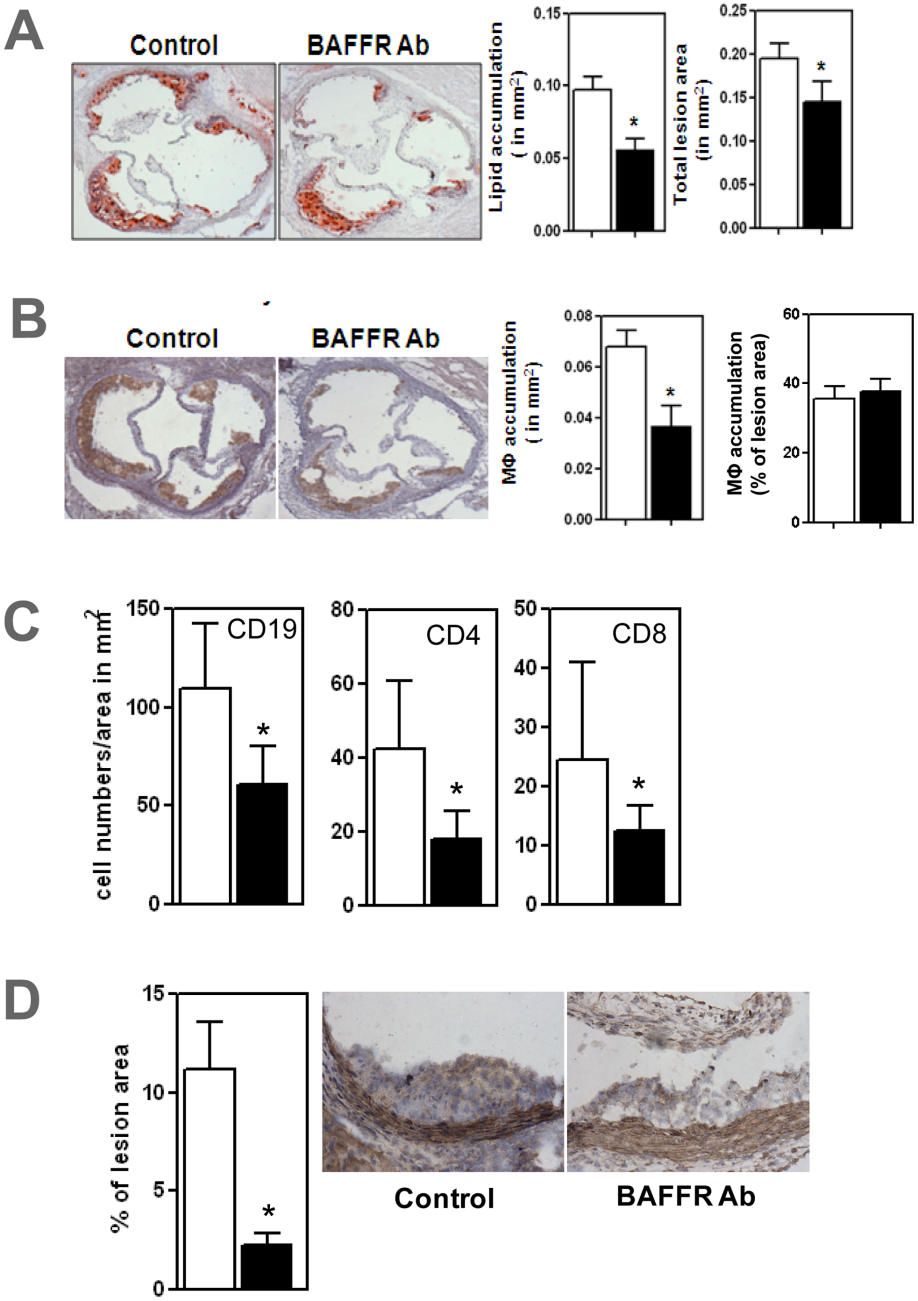
Atherosclerosis ameliorated in ApoE^−/−^ mice treated with anti-BAFFR antibody accompanied by immunohistochemical demonstration of reduced B cells, CD4^+^T cells, CD8^+^T cells and IL1β in aortic lesions. (A) Oil Red-O stained lipid accumulation and total lesion area at aortic roots (B) CD68+ macrophage accumulation. Immunohistochemical analysis show decreased CD19^+^ B cells, CD4^+^ T cells, CD8^+^ T cells (C) and IL1β (D) in atherosclerotic lesions. n = 7–9 mice per each group * p<0.05 □ control IgG treated mice; ▪ BAFFR-antibody treated mice.

Total atherosclerotic lesion areas in the BAFFR antibody treated mice decreased by 25% compared to those in the control group ([P<0.05]; Figure 3A). Lipid accumulation assessed by oil red-O staining and macrophage accumulation, assessed by CD68 antibody staining were reduced by 36% and 46% respectively in ApoE^−/−^ mice ([P<0.05]; Figures 3A–B). Immunohistochemical analysis revealed that CD19^+^ B cells, CD4^+^ T cells and CD8^+^ T cells were reduced by 41%, 55% and 50% respectively in atherosclerotic lesions of anti-BAFFR-antibody treated ApoE^−/−^ mice compared to control group ([all P<0.05]; Figure 3C). Proinflammatory cytokine IL1β were decreased by 60% in the atherosclerotic lesions ([P<0.05]; Figure 3D).

### Decreased Arterial Inflammation in BAFFR-antibody-treated ApoE^−/−^ Mice

**Figure 4:**
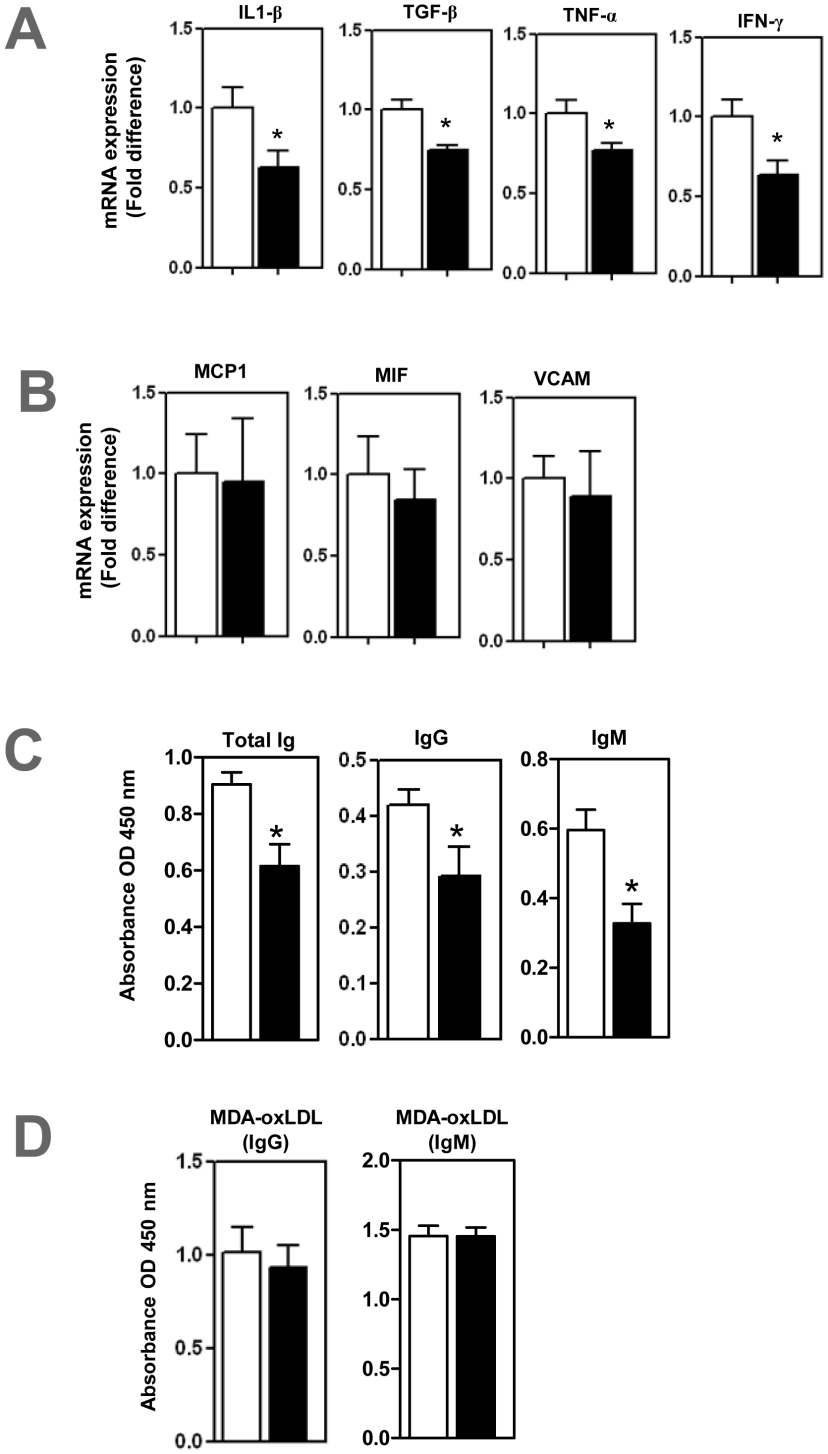
Reduced aortic inflammation and plasma immunoglobulins in anti BAFFR antibody treated- ApoE^−/−^ mice. Real-time PCR analysis shows (A) Cytokines- IL1β, TGFβ, TNFα and IFNγ- decreased, but (B) MCP1, MIF and VCAM were unaffected. ELISAs show (C) plasma total immunoglobulins decreased and (D) MDA-oxLDL specific antibodies unaffected. n = 7–9 mice per each group * p<0.05 □ control IgG treated mice; ▪ BAFFR-antibody treated mice.

Real-time PCR analysis revealed that proinflammatory cytokines IL1β, TGFβ, TNFα and IFNγ were reduced by 37%, 25%, 23% and 36% respectively in anti-BAFFR antibody treated mice compared to control mice ([all P<0.05]; Figure 4A). However, expressions of MCP1, MIF and VCAM-1 were unaffected in the BAFFR antibody treated mice (Figure 4B).

### Immunoglobulin Production in BAFFR-antibody Treated ApoE^−/−^ Mice

The finding that BAFFR antibody selectively depletes B2 B cells without affecting peritoneal B1a cells prompted us to determine effects on the plasma levels of total antibodies and MDA-LDL specific antibodies. ELISA determination showed that plasma levels of immunoglobulins (total, IgG and IgM) were reduced by 32%, 31% and 45% respectively in anti-BAFFR antibody treated ApoE^−/−^ mice ([all P<0.05]; Figure 4C). However we did not find any difference in MDA-oxLDL specific immunoglobulins (IgG and IgM) imposed by anti-BAFFR antibody treatment (Figure 4D).

### Therapeutic Application of Anti-BAFFR Antibody Reduces Mature B Cells in ApoE^−/−^ Mice

**Figure 5:**
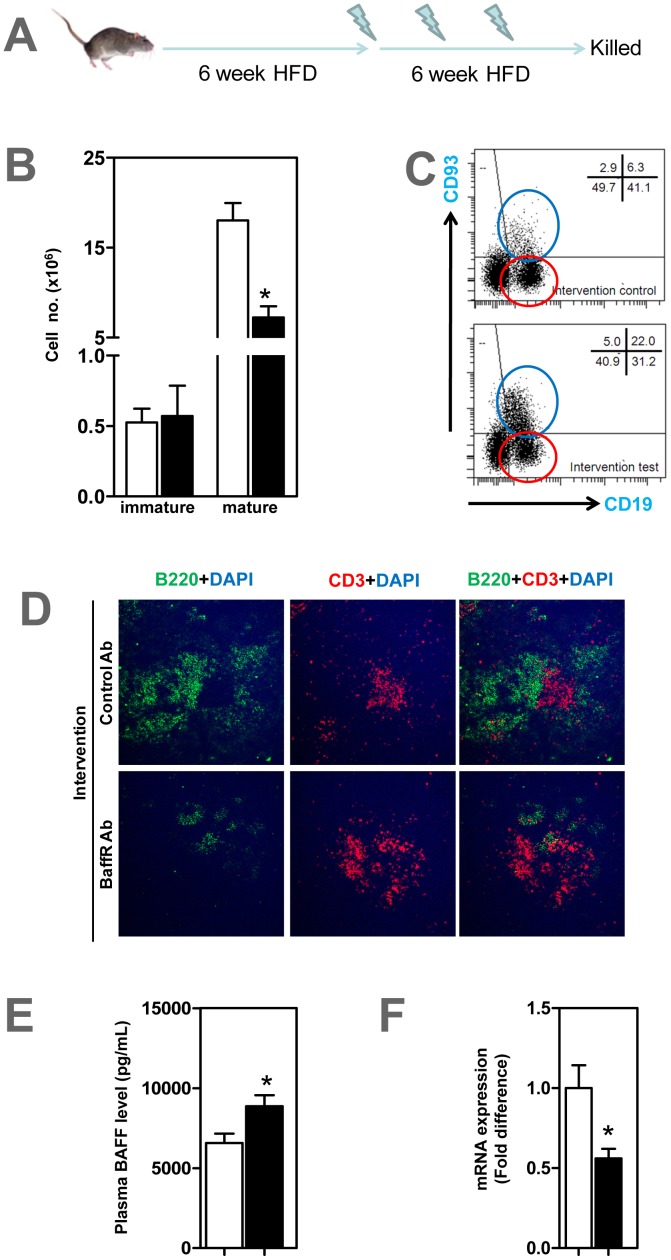
Anti-BAFFR antibody selectively depletes mature B cells, not B1a cells in ApoE^−/−^ mice with established atherosclerosis. (A) ApoE^−/−^ mice fed a HFD for 6 weeks to generate atherosclerosis, followed by three doses of anti-BAFFR antibody while feeding a HFD for a further 6 weeks. At end of experiment, FACS analysis show (B-C) mature B2 B cells in spleens selectively depleted. Representative FACS image show (C) mature and immature B cells in spleen stained with CD93 and CD19 antibodies Blue circle represents CD93^+^ CD19^+^ immature B cells and red circle CD93^−^ CD19^+^ mature B cells. (D) Spleen B cell zones disrupted in BAFFR-depleted mice compared to control group. (E) Plasma levels of BAFF determined by ELISA increased in anti-BAFFR-antibody treated ApoE^−/−^ mice. (F) mRNA expression of CD20 in spleens reduced in BAFFR-treated mice. n = 7–9 mice per each group * p<0.05 □ control IgG treated mice; ▪ BAFFR-antibody treated mice.

Since BAFFR antibody treatment attenuated development of atherosclerosis, we next examined how treatment would affect progression of already developed atherosclerotic lesions. To experimentally simulate clinical presentation of atherosclerosis in ApoE^−/−^ mice, we fed the mice a HFD for 6 weeks to establish atherosclerosis. We then treated these mice with anti-BAFFR antibody while feeding them a HFD for a further 6 weeks (Figure 5A). FACS analysis showed that mature B cells were depleted in spleen ([P<0.05]; Figures 5B–C) and blood (data not shown) in anti-BAFFR antibody treated mice. Spleen B cell zones were also disrupted with loss of B220^+^ B cells (Figure 5D), plasma BAFF levels were increased by 27% ([P<0.05]; Figure 5E) and CD20 expression in spleen was reduced by 44% ([P<0.05]; Figure 5F) in anti-BAFFR antibody treated ApoE^−/−^ mice, as in the development study (see Figure 1).

### Anti-BAFFR Antibody Reduces Progression of Established Atherosclerosis in ApoE^−/−^ Mice

**Figure 6:**
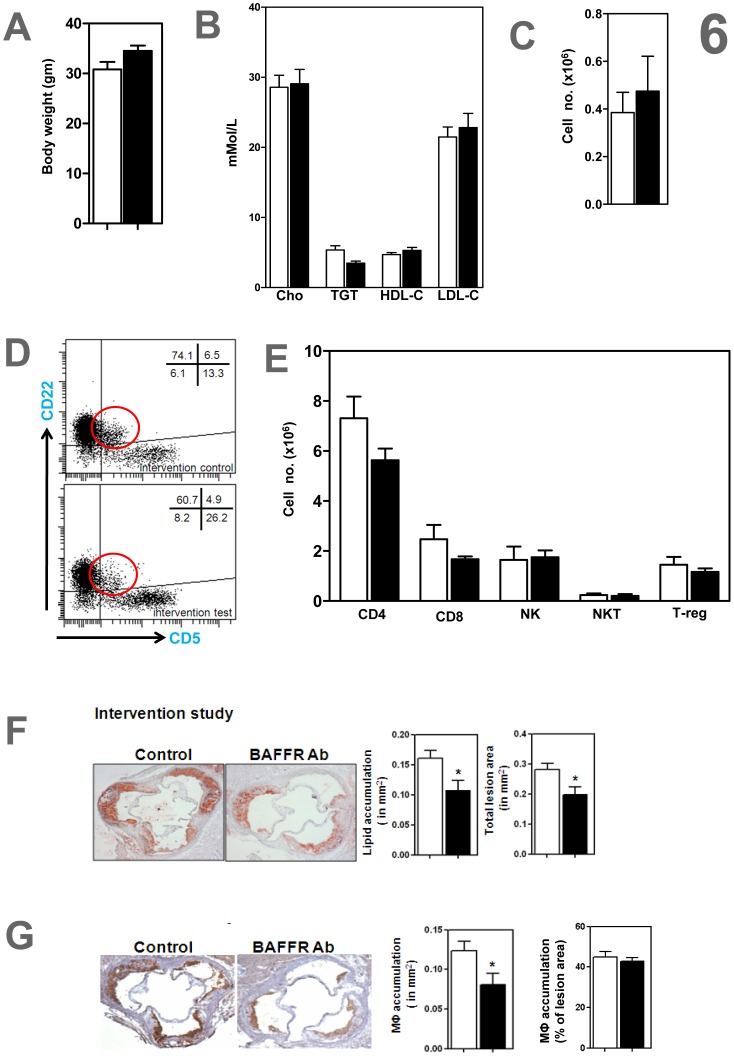
Anti-BAFFR antibody reduced atherosclerosis in hyperlipidemic ApoE^−/−^ mice with established atherosclerosis. Despite (A) body weight and (B) comparable plasma lipids, anti-BAFFR antibody ameliorate atherosclerosis in ApoE^−/−^ mice with established atherosclerosis assessed by (F) Oil Red-O stained lipid accumulation and total lesion area and (G) CD68^+^ macrophage accumulation. (C-D) peritoneal B1a B cells unaffected, representative FACS image show that CD22^+^ CD5^+^ peritoneal b1a cells were unaffected. (E) CD4^+^, CD8^+^, NK1.1^+^ (NK) and NK1.1^+^ TCRβ^+^ (NKT) cells and CD4^+^ CD25^+^ foxp3^+^ regulatory T cells (T-reg) in spleens unaffected n = 7–9 mice per each group * p<0.05 □ control IgG treated mice; ▪ BAFFR-antibody treated mice.

Body weight and, plasma LDL-Cholesterol, HDL-Cholesterol, total cholesterol and triglycerides were unaffected in anti-BAFFR antibody treated ApoE^−/−^ mice (Figures 6A–B). FACS analysis showed that peritoneal B1a cells (Figures 6C–D) and other lymphocyte populations in the spleens (Figure 6E) remained unchanged in the anti- BAFFR-antibody treated mice. Anti-BAFFR antibody treatment of atherosclerotic ApoE^−/−^ mice indicated attenuated progression of atherosclerosis, with reductions in atherosclerotic lesions, by 30% (total lesion size), 33% in oil red-O stained lipid accumulation and 35% in CD68+ macrophage accumulation ([P<0.05]; Figures 6F–G). The observations that macrophage accumulation expressed as a percentage of total atherosclerotic lesion area was unaffected by anti-BAFFR-antibody (Figures 3B, 6G) suggest that the reduced macrophage staining reflects smaller plaques.

## Discussion

B2 B cells are important proatherogenic lymphocytes known to promote development and progression of atherosclerosis^3^. Our findings using an anti-BAFFR monoclonal antibody indicate that specific targeting this B cell subtype is a potentially important therapeutic strategy to prevent both development and also progression of already established atherosclerotic lesions to more complex lesions that may lead to heart attacks and strokes. Anti-BAFFR antibody treatment appears to affect only the B2 B cell population with B1a B cells and other lymphocyte populations being unaffected. Its effects on atherosclerosis are independent of plasma lipid concentrations.

B cell homeostasis is balanced by the numbers of B cells and circulating cytokine BAFF levels. Plasma BAFF level is indirectly proportional to circulating B cells indicating a negative feedback homeostatic mechanism [22]. Increased plasma BAFF levels in anti-BAFFR antibody treated ApoE^−/−^ mice is in agreement with mature B cell depletion where we showed decreased mature B2 cells, decreased mRNA expression of CD20 and disrupted B-cell zones in the spleen. Our findings are in accord with the report that soluble BAFF levels are inversely correlated with peripheral B cell numbers and the expression of BAFF receptors [23]. The findings are also consistent with the report of increased plasma BAFF levels in patients with autoimmune disorders treated with anti-CD20 monoclonal antibody rituximab [24], [25].

Macrophage attractant protein (MCP1) and the adhesion molecule VCAM-1 have been ascribed a role in recruiting leukocytes into developing atherosclerotic lesions [26]. In contrast to our previous report where MCP1 and VCAM-1 expression were reduced in BAFFR^−/−^ ApoE^−/−^ mice [12], anti-BAFFR antibody did not affect MCP1 and VCAM-1 expression in the anti-BAFFR antibody treated mice. These differing results may reflect the difference between long-term depletion of BAFFR imposed by genetic knock-out versus short-term blockade of BAFFR by the monoclonal antibody. However, in agreement with our previous report where a mature B2 deficient environment arising from genetic BAFFR knockout generated less infiltrating lymphocytes into atherosclerotic lesions [12], we also found less CD4^+^ and CD8^+^ T cells in the anti-BAFFR antibody treated ApoE^−/−^ mice. Given that B2 cells are professional antigen presenting cells (APCs) that can present antigen to CD4^+^ T cells and cross-present to CD8^+^ T cells [27], depletion of these B2 cells may be responsible for the reduced infiltration of CD4^+^ and CD8^+^ T cells into atherosclerotic lesions. Reduced infiltration of these T cells into aortic lesions may have contributed to the reduction in atherosclerotic lesions. Indeed transfer of CD4^+^ T cells to immunodeficient mice have been reported to aggravate atherosclerosis development [28]. Further, antigen presentation by APCs to CD4^+^ T cells in the arterial wall has been reported to cause local T cell activation and production of proinflammatory cytokines that promote atherosclerosis by maintaining chronic inflammation and induction of foam cell formation [29].

Proinflammatory cytokines produced in atherosclerotic lesions contribute to local inflammatory responses and progression to unstable atherosclerotic plaques. There is increasing recognition of cytokines produced by B cells having a key role as regulators of immunity, especially in local inflammatory responses [30]. Indeed B cells produce different cytokines, depending on their environment, to modulate local immune responses [30]–[32]. As well other immune cells that have infiltrated the atherosclerotic plaque such as macrophages, CD4^+^ and CD8^+^ T cells also produce proinflammatory cytokines [33]–[35]. These cytokines contribute towards local inflammation and may act on their own cells in an autocrine fashion or neighbouring cells in a paracrine fashion to further enhance production of proinflammatory cytokines [36] to promote chronic inflammation. Our results showed that a significant reduction of the proinflammatory cytokine IL1β as detected by both protein and mRNA expression in atherosclerotic lesions was observed in anti-BAFFR antibody treated mice. The reduced IL1β may have contributed to the reduced atherosclerosis, given that a genetic deletion of IL1β decreases the severity of atherosclerosis in ApoE^−/−^ mice [37]. Macrophages, the most prominent immune cell within atherosclerotic lesions are responsible for producing many of the proinflammatory cytokines. Macrophages and IL1β are found in all stages of atherosclerosis development and contribute to local inflammatory responses [38]. As well as reduced IL1β expression, TNFα, TGFβ and IFNγ were also reduced in anti-BAFFR antibody treated mice. The reductions in TNFα and IFNγ may also have contributed to the reduced atherosclerosis IL1β and TNFα enhance macrophage-derived foam cell formation by inhibiting macrophage intracellular lipid catabolism [39]. Also, atherosclerosis is reduced in ApoE^−/−^ mice deficient in TNFα [40] and IFNγ potentiates atherosclerosis in these mice [41].

Our findings of reductions in immunoglobulins in anti-BAFFR antibody treated mice is consistent with mature B2 depletion and with reports of their reduction in BAFFR^−/−^ and BAFFR^−/−^ ApoE^−/−^ mice [12], [42] because BAFF-BAFFR interaction is required in isotype-switching and enhances antibody production [43], [44]. However, we did not find any difference in MDA-oxLDL specific immunoglobulins in anti-BAFFR antibody treated mice. This may reflect disruption in the B cell zone within the spleen and the reduction in mature B2 cells with consequent disruption of B cell development into plasma cells that produce antigen-specific immunoglobulins after antigen exposure.

### Conclusion

In summary, we have shown that anti-BAFFR antibody treatment in hyperlipidemic ApoE^−/−^ mice not only prevented atherosclerosis development but also attenuated the progression of established atherosclerosis. Mature B cells and proinflammatory cytokines implicated in atherosclerosis pathogenesis were decreased together with the ameliorated atherosclerosis in the anti-BAFFR antibody treated ApoE^−/−^ mice. As a B cell depletion therapeutic strategy for atherosclerosis, anti-BAFFR monoclonal antibody seems therapeutically more specific than anti-CD20 monoclonal antibody treatment as it only depletes atherogenic B2 cells while sparing atheroprotective B1a cells. It is tempting to speculate that combined with a lipid-lowering strategy, anti-BAFFR antibody treatment has potential to even more effectively reduce progression of atherosclerosis and reduce the lethal complications of atherosclerosis-related myocardial infarction and stroke.
